# The Invisible Frontline: High‐Tech Root Imaging for Crop Stress Adaptation

**DOI:** 10.1111/ppl.70572

**Published:** 2025-10-15

**Authors:** Rahul Chandnani, Raju Soolanayakanahally

**Affiliations:** ^1^ Saskatoon Research and Development Centre Agriculture and Agri‐Food Canada Saskatoon Saskatchewan Canada

**Keywords:** image analysis, rhizotrons, root phenotyping, root system architecture, x‐ray‐computed tomography

## Abstract

Roots are crucial for enhancing crop resilience to abiotic stresses, including drought, salinity, cold, nutrient deficiency, and metal toxicity. Root system architecture and morphological traits play a significant role in enabling plants to access water and nutrients under stress conditions. However, the study of roots is challenging due to their underground nature. Here, we review advancements in high‐throughput root phenotyping methodologies that enable the non‐destructive and large‐scale analysis of root traits in controlled conditions. These include soil‐less two‐dimensional platforms, such as hydroponics and gel‐based systems, and soil‐based systems like Rhizotrons and RhizoTubes. Additionally, cutting‐edge three‐dimensional soil‐less systems and soil‐based imaging technologies, such as x‐ray‐computed tomography and magnetic resonance imaging, have significantly improved the precision of root trait analysis. Computational tools, including machine learning algorithms, are also transforming root phenotyping by automating image segmentation, trait extraction, and data analysis. Case studies and examples described here demonstrate the successful application of these methods in identifying stress‐specific root traits that improve resilience to various abiotic stresses in monocots, dicots, and legumes. Despite these advancements, challenges such as high costs, scalability, and environmental variability persist. Integrating laboratory and field‐based phenotyping systems can address these limitations and lead the way for more effective breeding programs to improve crop resilience against climate change.

## 1 Introduction

The global challenge of ensuring food security is increasingly threatened by abiotic stresses that adversely impact crop production. Abiotic stresses, which include drought, salinity, cold, nutrient deficiency, and metal toxicity, significantly diminish crop yields and quality, ultimately affecting the global food supply (Basso and Ritchie 2014; Carr et al. 1991; Füllgrabe et al. 2022; Haq et al. 2014; Kang et al. 2011; J. Lynch 1995; Shimizu et al. 2004; Shimono 2011; Vadez et al. 2013; Zörb et al. 2019). These stresses can result in substantial economic losses, particularly in regions that are heavily reliant on agriculture. Understanding the mechanisms by which crops respond to various stresses is critical for the development of resilient agricultural practices. This literature review investigates and summarizes advanced methodologies for analyzing root traits to enhance crop resilience. Agricultural crops are constantly exposed to a range of abiotic stresses, which, whether individually or in combination, can severely compromise their growth and productivity (Mittler 2006). For instance, drought stress alone accounts for greater annual crop losses than any other abiotic factor (Boyer 1982). Studies have demonstrated that drought conditions can significantly reduce yields in barley, maize, and rice (Farooq et al. 2009). Salinity, another significant stressor, affects over 20% of irrigated land globally, resulting in diminished crop yields and reduced soil fertility (Munns and Tester 2008). Research has indicated that rice yields may decline by more than 60% under conditions of high salinity (Zheng et al. 2023).

Cold stress can adversely affect cellular structures and metabolic processes, particularly in crops that are not acclimatized to low temperatures. This condition results in diminished germination rates, stunted growth, and reduced yields (Thakur et al. 2010; Thomashow 1999). For instance, cold stress has been documented to significantly decrease the yield of maize (Badu‐Apraku et al. 1983). Nutrient deficiency represents another critical concern. Essential nutrients such as nitrogen, phosphorus, and potassium are imperative for crop growth and development; their deficiency may lead to suboptimal growth, chlorosis, and a marked decrease in yields (Tan et al. 2005). Noulas et al. (2018) emphasized that zinc deficiency alone impacts almost half of the global cereal‐growing regions, resulting in considerable yield reductions. Additionally, metal toxicity, induced by elements such as aluminum and cadmium, can inhibit root growth and nutrient uptake, further stressing the plants (Hou et al. 2019; Kochian et al. 2004).

Root system architecture (RSA) plays a crucial role in determining a plant's capacity to access water and nutrients in stressful conditions. Variations in RSA characteristics, including root depth, angle, and branching patterns, significantly influence a plant's resilience to abiotic stresses (J. P. Lynch 2007). For example, crops with deeper and more extensive root systems are better prepared to endure drought conditions (Comas et al. 2013). Similarly, root traits that promote efficient nutrient uptake are essential for managing nutrient deficiencies (Lynch and Brown 2012). Uga et al. (2013) have demonstrated that rice varieties with deeper rooting systems exhibit enhanced drought tolerance. Moreover, research conducted by Bates and Lynch (2000) highlights the significance of root hair length and density in phosphorus acquisition under low‐phosphorus conditions. Additionally, Trachsel et al. (2013) indicated that a deeper root angle plays a crucial role in nitrogen uptake efficiency in maize. These variations in RSA traits not only determine a plant's capacity to adapt to various abiotic stresses but also contribute to improved crop productivity and resilience. Investigations by Vaughan et al. (2002) have demonstrated that a fibrous root system in Alfalfa is associated with improved tolerance to saline conditions. Such thorough analyses and findings highlight the critical role of specific RSA traits in enabling plants to thrive under adverse environmental conditions.

Certain RSA traits, including increased density of long lateral roots in maize, can enhance a plant's capacity to withstand cold stress by improving water and nutrient absorption during early seedling growth under unfavorable conditions (Hund et al. 2008). Research conducted by Berry et al. (2004) has elucidated how root architecture contributes to lodging resistance in wheat. Their findings emphasize that deeper and more robust root systems can significantly diminish the likelihood of lodging by providing enhanced anchorage and stability to the plants. Furthermore, a study conducted by Wu and Ma (2016) examined root architecture in canola and its impact on lodging resistance. The research indicated that canola varieties characterized by a denser root system and a greater root mass exhibited increased lodging resistance, thus providing critical insights for breeding programs focused on improving crop durability and productivity.

Moreover, investigating root systems presents inherent challenges due to their underground nature, which complicates direct observation and measurement (Rich and Watt 2013). Conventional methods of root study often involve labor‐intensive and destructive sampling techniques, thereby restricting their applicability for large‐scale research initiatives. This emphasizes the necessity for the development and application of high‐throughput phenotyping technologies to expedite root trait analysis and breeding methodologies (Topp et al. 2013).

High‐throughput root phenotyping (HTRP) methodologies have emerged as essential tools for the non‐invasive and large‐scale analysis of root traits. These technologies facilitate the detailed examination of RSA and other root characteristics under various stress conditions, thereby enabling the identification of stress‐resilient traits (Paez‐Garcia et al. 2015). For instance, hydroponic systems and gel‐based platforms have been utilized to investigate root responses to nutrient deficiencies. By integrating advanced imaging techniques and computational tools, HTRP platforms yield comprehensive data regarding root morphology, anatomy, and functionality (Mooney et al. 2012).

The amalgamation of high‐throughput root phenotyping technologies with conventional breeding methodologies presents considerable potential for augmenting crop resilience to abiotic stresses. By enabling a thorough investigation of root characteristics, these techniques can identify stress‐specific traits that bolster crop productivity in adverse conditions. Ongoing advancements in root phenotyping and the formulation of economically viable and scalable solutions will be imperative for addressing the challenges posed by climate change and ensuring global food security (Furbank and Tester 2011).

## 2 Overview of Root Traits Relevant to Abiotic Stress

### 2.1 Root Morphology Traits

**FIGURE 1:**
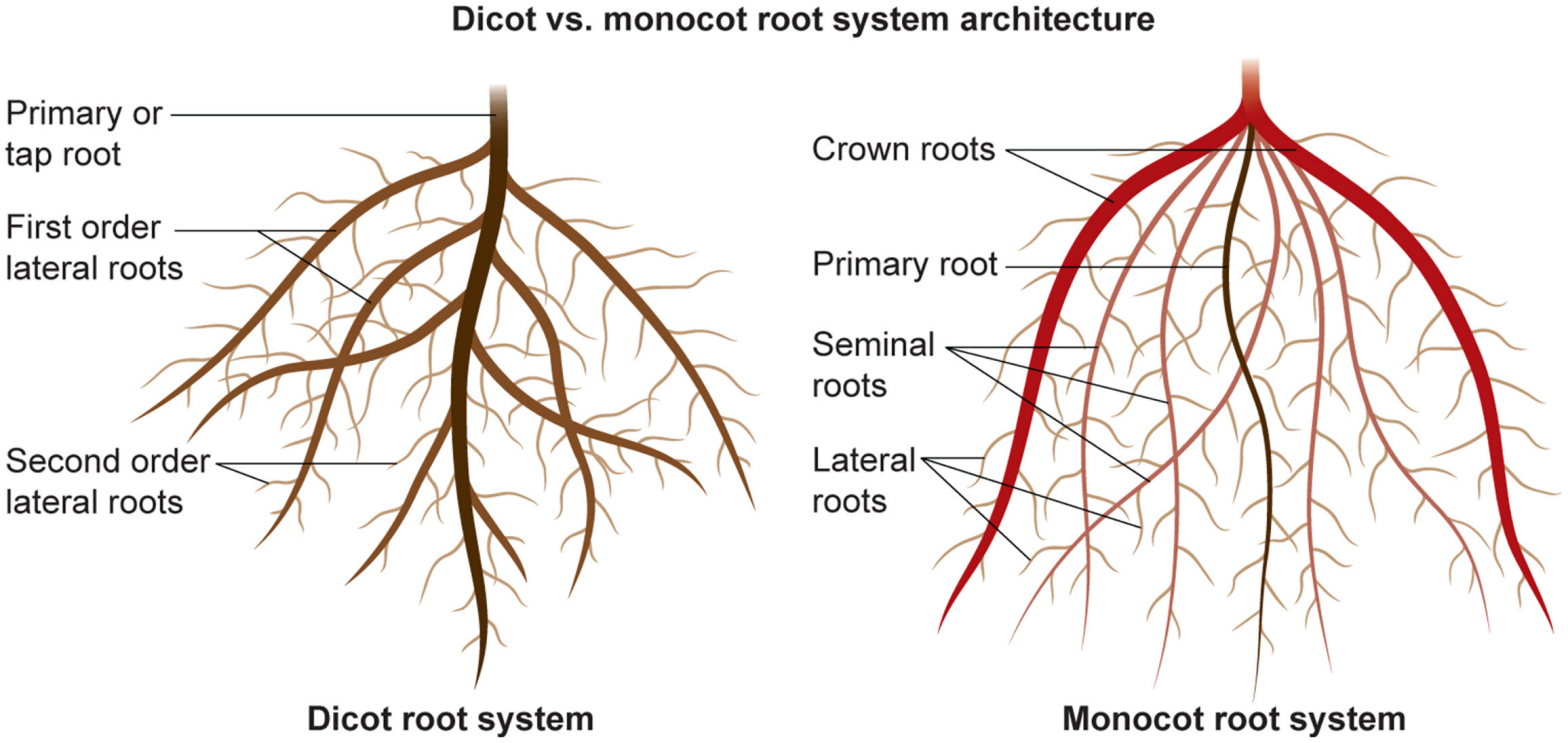
This figure illustrates the ideotype root structures and the various common and distinct components of the root systems in monocots and dicots.

**FIGURE 2:**
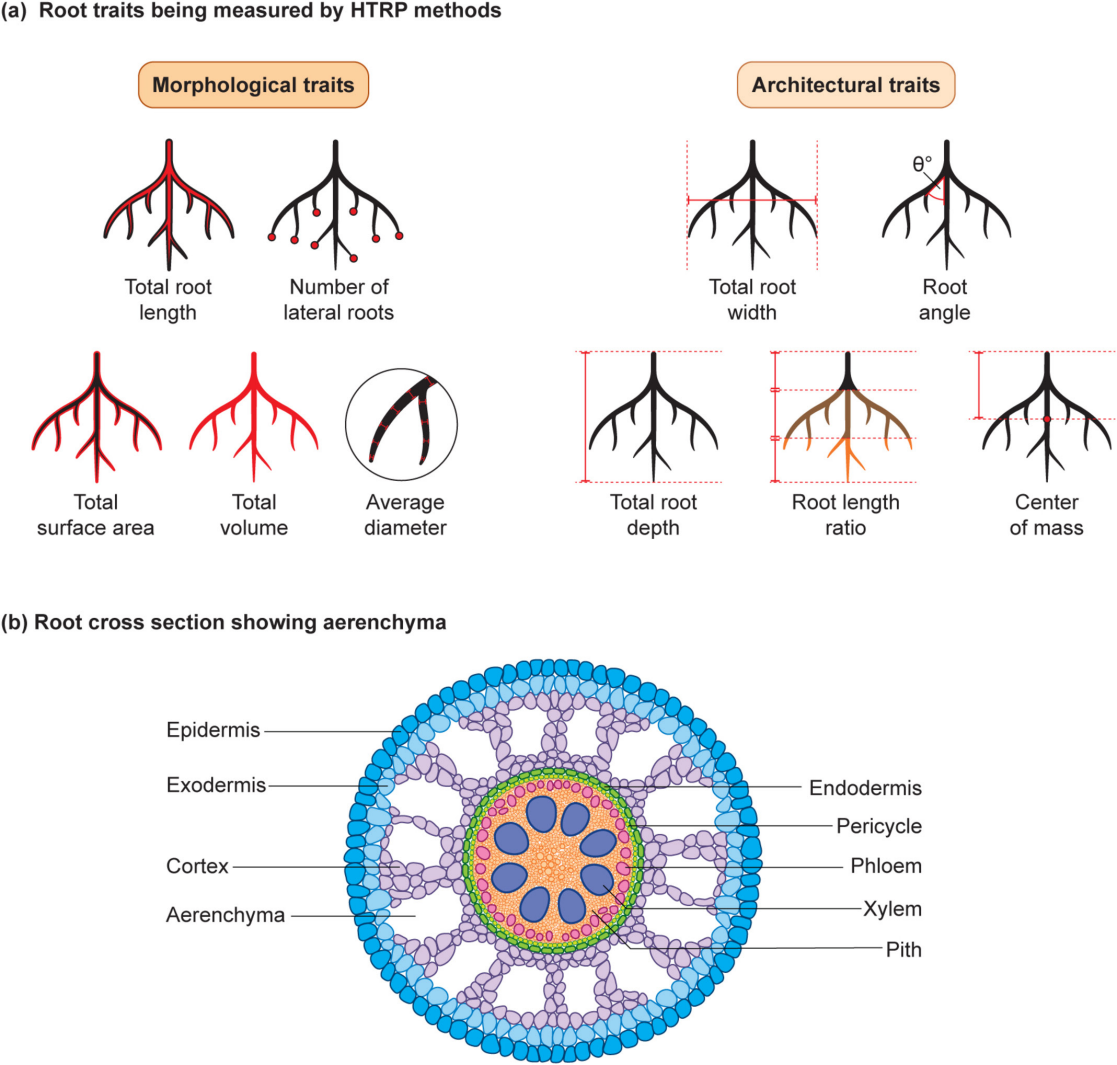
(a) Illustration of some of the most common morphological and architectural traits extracted from high‐throughput root phenotyping data. (b) Root cross‐section showing aerenchyma.

Monocot and dicot root systems differ inherently in their root morphological and architectural traits (Figure 1). Root morphology characteristics, including greater total root length and lateral root count, enhance nutrient absorption and stress resilience (Figure 2a). Research by Jia et al. (2018) indicated that increased lateral root branching and higher root length density within the top 20 cm of soil (with many short ideotypes) significantly improved phosphorus uptake in low‐phosphorus soils, resulting in an 18% higher yield than fewer longer ideotypes. Conversely, a greater number of lateral roots correlates positively with increased yield in water‐limited environments for soybean (Prince et al. 2019). Furthermore, an increase in metaxylem numbers enhances hydraulic conductivity and drought tolerance in soybean (Prince et al. 2017). Chimungu et al. (2014) found that larger cortical cells in maize roots decrease metabolic costs, facilitating deeper root penetration to access lower soil layers and maintain water absorption during drought. Additionally, root volume is a vital trait for abiotic stress resilience. Chu et al. (2014) demonstrated that higher root biomass and root length density in rice improve salinity stress tolerance. Root hairs are tubular extensions of root epidermal cells that enhance water and nutrient uptake. Basic helix–loop–helix proteins regulate their development and may be influenced by environmental stresses, such as drought, prompting adaptive modifications in length and density to optimize resource acquisition (Kohli et al. 2022; Wang et al. 2024). In phosphorus deficiency, root hairs grow longer to enhance rhizosphere exploration. This has been demonstrated in common bean genotypes using a simple germination paper method, revealing genetic differences in hair density and length (Vieira et al. 2007). Hormonal pathways involving auxin and ethylene regulate initiation, with RSL genes serving as stress regulators that facilitate microbial recruitment for tolerance (Vissenberg et al. 2020). While macro‐tools overlook them, micro‐CT can capture traits with high resolution (Duddek et al. 2022). Brace roots, also known as nodal adventitious roots, emerge from aerial stem nodes in maize and cereals. They provide structural support to prevent lodging and contribute to water and nutrient uptake during later developmental stages (Sparks 2023). Research from Erin Sparks' lab at the University of Delaware explores their biomechanics and genetics, assessing properties across whorls, genotypes, and growth stages to reveal variations in bending moduli and geometry that influence plant stability (Hostetler et al. 2022). These roots respond to signals such as gravity and displacement, reinforcing anchorage. Genetic studies have also identified genes and QTLs linked to brace root traits, paralleling findings in sorghum, to develop lodging resistance (Reneau et al. 2024).

### 2.2 Root Architecture and Anatomical Traits

Steeper root growth angles and deeper root systems (Figure 2a) have been shown to enhance drought tolerance and nitrogen deficiency tolerance by accessing deeper soil moisture, ensuring that plants receive adequate water even under dry conditions (J. P. Lynch 2022).

The DEEPER ROOTING 1 (DRO1) allele present in rice lines results in steeper and deeper nodal roots, enhancing their ability to withstand drought stress (Uga et al. 2011, 2013). The angle of root growth affects foraging in both topsoil and subsoil in common bean; deeper angles facilitate better exploration of subsoil and enhance water capture, thus increasing drought resistance (Ho et al. 2005). In wheat, a steeper growth angle of nodal roots correlates with improved access to deep soil water, resulting in higher yields during drought (Manschadi et al. 2010). Maize lines with steeper root growth angles exhibit significantly better root depth, N capture, and overall plant performance under N stress in greater precipitation areas, whereas shallow root systems perform well in drier regions (Dathe et al. 2016; Trachsel et al. 2013). A greater nodal root growth angle in a mutant maize line corresponds to steeper angles at two nodal positions, leading to better N acquisition from deeper soil and enhanced growth in low‐N field conditions (Schneider et al. 2022).

Wider root systems can enhance nutrient uptake efficiency. Pinthus (1967) found that wider root angles and broader root spread in wheat increase resistance to lodging. Additionally, a top‐soil foraging approach combined with a shallower root system can increase phosphorus uptake in low‐phosphorus soil in maize (Zhu and Lynch 2004). The angle of the roots is vital for optimizing water and nutrient acquisition, as it largely determines whether root systems spread deeper or shallower. In a study by J. P. Lynch (2013), steeper root angles in maize were shown to help plants more effectively utilize soil resources. Lynch's research studies have demonstrated that steeper root angles facilitate deeper soil penetration, thereby improving access to water and nitrogen. Manschadi et al. (2006) highlighted the essential roles of root length distribution and architecture in enhancing cereal resilience across diverse soil conditions. Furthermore, alterations in root anatomical characteristics have also played a role in enhancing abiotic stress tolerance by modifying root morphology and architecture through the manipulation of carbon cost. For instance, the increased formation of root cortical aerenchyma in maize results in reduced lateral root branching accompanied by elongated axial roots, a modification that has been associated with improved nitrogen uptake (Figure 2b).

### 2.3 Emerging Proxies for Functional Root Traits Beyond Structural Measurement

While HTRP systems excel at measuring structural characteristics like root length, angle, and depth, they find it more challenging to quantify functional traits such as water uptake efficiency, exudation profiles, and ion transport because these are often dynamic and depend on environmental context. Recent methods incorporate isotopic techniques, sensor technologies, and microbiome mapping to better assess these traits, advancing the field toward a more complete understanding of root functionality.

Isotopic labeling serves as a proxy for water uptake efficiency by using stable isotopes like deuterium oxide to trace water sources in the soil. When paired with natural abundance methods, this technique allows for the quantification of deep root contributions during droughts, with deep‐rooted varieties showing up to three times higher uptake values. In a study by Guo and Zhao (2021) in alley cropping systems, δ^2^D and δ^18^O labeling were employed to model water uptake, revealing genotypic variations in water uptake efficiency. Root exudate sensors detect exudation profiles essential for nutrient mobilization and attracting microbes. A microfluidic device with colorimetric sensors allows for continuous, spatial monitoring of exudates along roots, showing how they change over time (Patko et al. 2024). Spectroscopic techniques detect dissolved organic carbon in exudates, linking profiles to plant stress responses. Mapping the rhizosphere microbiome involves proxy measures of ion transport and root function by analyzing microbial genes related to nutrient processing. Recent research aims to identify the genetic factors involved in microbiome assembly, including QTLs that influence ion‐related microbes (Escudero‐Martinez et al. 2022).

## 3 High‐Throughput Root Phenotyping Technologies

### 3.1 Soilless and Transparent Soil Systems

#### 3.1.1 2D Root Phenotyping

##### 3.1.1.1 Free‐Floating Hydroponic Systems

**FIGURE 3:**
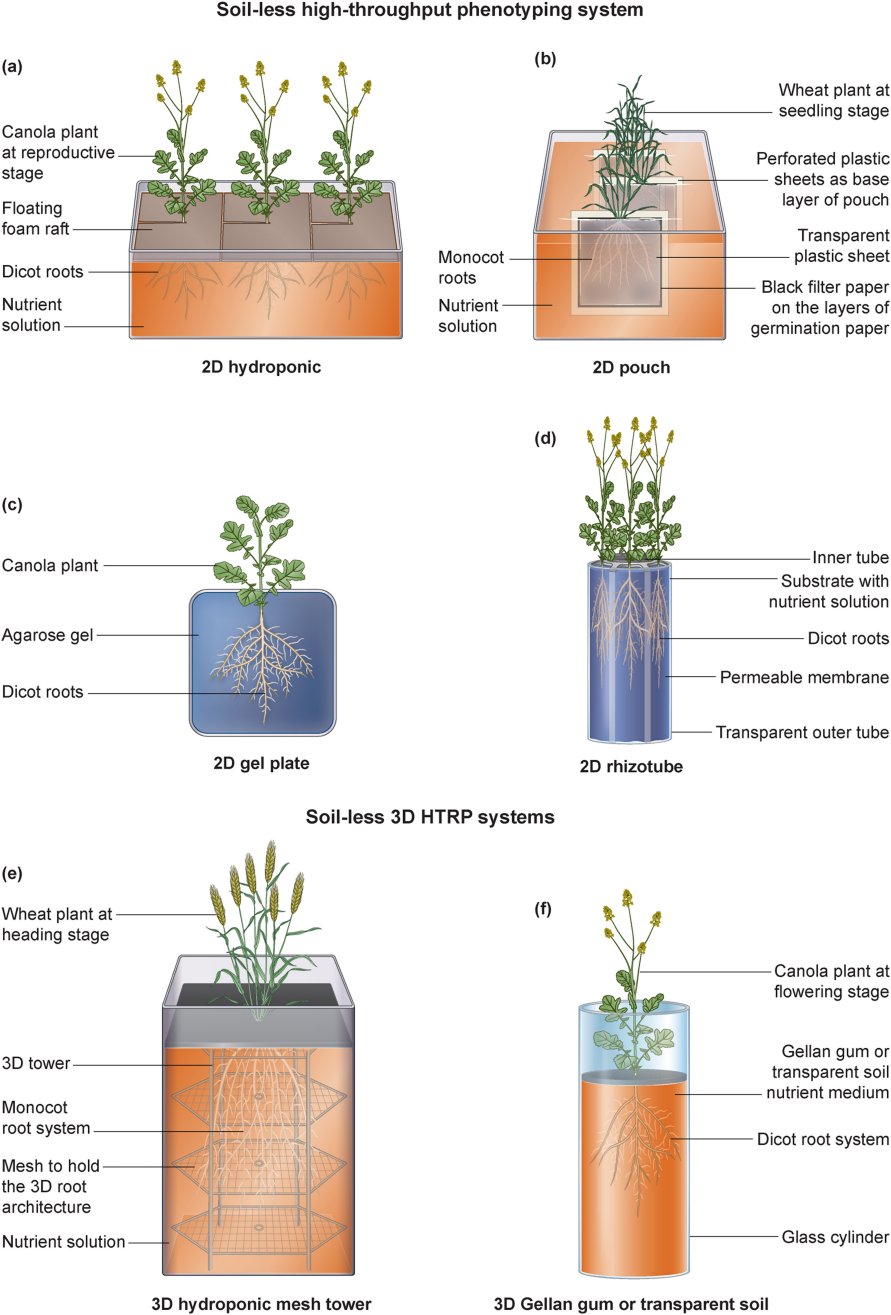
(a) Illustration of canola plants at the reproductive stage within a free‐floating hydroponic root growth system. (b) Illustration of wheat plants during the seedling phase in a two‐dimensional pouch system. (c) Illustration of a canola plant at the seedling stage in an agar gel plate root growth system. (d) Illustration of canola plants at the reproductive stage utilizing a two‐dimensional RhizoTube root growth system. (e) Illustration of a wheat plant cultivated in a three‐dimensional hydroponic mesh tower system. (f) Illustration of a canola plant growing within a three‐dimensional Gellan gum system.

These systems suspend plants in nutrient solutions, allowing for precise control over root zone conditions (Figure 3a). Research on cereal crops has demonstrated their utility for early seedling and later‐stage root screening in free‐floating hydroponic solutions (Clark et al. 2013; Famoso et al. 2010; Li et al. 2012, 2007; Ni et al. 1998; Panigrahy et al. 2014; Ren et al. 2012). Although widely applied for investigating nutrient uptake and stress responses, their limited mechanical impedance does not accurately mimic natural soil environments. Studies such as Ni et al. (1998) have mapped the pup1 QTL in hydroponics. Near‐isogenic lines with the effective Pup1 allele demonstrated a threefold increase in phosphorus uptake and grain yield under low‐phosphorus conditions compared to the recurrent parent, cv Nipponbare, which is a line sensitive to phosphorus deficiency (Wissuwa and Ae 2001). Similarly, Wang et al. (2017) illustrated how insights from seedling growth stage screenings of maize plants overexpressing protein phosphatase 2A regulatory subunit A, ZmPP2AA1, translated into enhanced maturity‐stage yield traits under phosphorus deficiency. Observations included increased root dry weight, proliferating lateral root branching, and an elevated root‐to‐shoot ratio in the overexpressed plants. Likewise, a free‐floating hydroponic high‐throughput root phenotyping system was initially utilized for the identification (Maron et al. 2008) and later functional characterization (Maron et al. 2010) of the aluminum tolerance gene MATE1 in maize.

The rolled paper towel method is an affordable, soil‐free technique used to evaluate early maize seedling growth and root traits under controlled conditions, such as hormone or stress treatments (Draves et al. 2022). Research work exhibited by Dior Kelley's lab at Iowa State University involves sterilizing seeds, preparing solutions, rolling seeds in towels, and growing them vertically for 7–10 days, then imaging to measure traits like length, branching, and gravitropism. Its advantages include high scalability, uniform treatment application, and suitability for genetic or chemical screening, though it may result in uneven moisture distribution and is limited to juvenile stages without soil mimicry (Draves et al. 2022). This method can be used to study auxin carriers like ZmPILS6, diversity panels, and dose responses to analogs.

##### 3.1.1.2 2D Pouches and Gel Plates

Transparent pouches and gel‐based media facilitate root observation on a fixed plane, allowing for image‐based quantification of traits such as elongation rate and lateral root formation (Figure 3b,c). Both pouches and gel plates function as sandwich systems, differing in that pouches contain a middle layer of germination paper or fabrics, while gel plates have agar gel sandwiched between glass or plastic plates. The 2D pouches and gel plates are particularly effective for early developmental studies in crops like cereals and soybeans (Chandnani et al. 2023; Falk et al. 2020; Hund et al. 2009; Pound et al. 2013; Richard et al. 2015). Although these systems can only be applied to the seedling stage, the traits have been validated to later stages of plant growth in a few of these cases. For example, Chandnani et al. (2023) confirmed a subset of root morphological traits obtained from the 2D pouch system at the pre‐flowering stage of soybean plants and experienced significant correlation in the variation of total root length, volume, and biomass among the soybean lines identified through the 2D pouch system. Notably, in addition to significant differences among genotypes, the root traits matched those varying between high‐yielding and low‐yielding soybean lines in a different field experiment (Prince et al. 2019). Notably, 2D gel plates have been widely utilized to identify root architecture genes in the model plant *Arabidopsis*, enhancing our understanding of the gene regulatory network that governs root development and architectural definition (Bengough et al. 2004; Chen et al. 2009; Julkowska et al. 2017; Stanga et al. 2009; Svistoonoff et al. 2007).

##### 3.1.1.3 RhizoTubes

The RhizoTubes system, developed by Jeudy et al. (2016), serves as a root phenotyping apparatus designed for high‐resolution, non‐destructive imaging of root development. This system comprises two concentric tubes: a rigid inner tube and a transparent outer tube, which are separated by a thin semi‐permeable membrane (Figure 3d). This membrane facilitates the passage of water, nutrients, and microorganisms while effectively obstructing root passage. Consequently, roots develop along the transparent wall, allowing for enhanced visualization. This configuration yields digital data that can be utilized to derive root characteristics such as length, angle, branching, and features of fine roots in real‐time. The RhizoTubes are applicable to a variety of plant species and are particularly beneficial for high‐throughput studies, especially under diverse abiotic stress conditions.

#### 3.1.2 3D Root Phenotyping

##### 3.1.2.1 3D Hydroponic Systems

Piñeros et al. (2016) designed 3D hydroponic systems that enable plant roots to grow freely in every direction, surpassing the limitations found in traditional 2D root observation platforms (Figure 3e). These systems remove visual barriers and allow for natural root growth behaviors, including radial expansion and diverse branching angles. This design supports the root system in a natural 3D orientation, avoiding compression into a flat plane. They utilized cylindrical containers filled with nutrient solutions, integrated with supporting structures (perforated ABS disc tower) to maintain the spatial distribution of roots, which facilitates the capture of intricate root traits such as root cone angle and root system volume. While this platform is less time‐intensive than Gellan gum and transparent soil platforms (Clark et al. 2011; Iyer‐Pascuzzi et al. 2010), the plastic discs can obstruct part of the roots in 2D images and 3D reconstructions prior to analysis. Currently, these disc towers are being enhanced to thin wire mesh towers that are simpler to construct and feature smaller gaps in the captured root images compared to the ABS plastic perforated discs (personal communication with Dr. Leon Kochian).

##### 3.1.2.2 3D Gellan Gum and Transparent Soil Systems

A glass cylinder is filled with transparent growth medium containing gel‐like engineered substrates, such as Gellan gum (Figure 3f) (Clark et al. 2011; Iyer‐Pascuzzi et al. 2010) or Nafion (Downie et al. 2012). These materials imitate soil‐like physical resistance while allowing light to pass through. This setup enables root visualization through optical microscopy and time‐lapse imaging, which reveals root branching and growth dynamics under abiotic stress (Clark et al. 2013; Downie et al. 2012; Iyer‐Pascuzzi et al. 2010; Ma et al. 2019). While these platforms more closely mimic the physical properties of soil, they are still limited by the time and resources required to prepare the growth medium on a large scale and the computational demands associated with 3D reconstruction analysis from image data.

### 3.2 Hybrid Methods

#### 3.2.1 Basket Method

The basket method has been extensively employed to evaluate root growth angle (RGA) and the deep rooting ratio in rice (Kato et al. 2006; Uga et al. 2009, 2011). The deep rooting ratio, defined as the proportion of total roots that penetrate to the bottom of the basket, was quantified to assess the extent of deep rooting (Uga et al. 2009). For quantitative trait locus (QTL) analysis of the ratio of deep rooting (RDR), open stainless‐steel mesh baskets measuring 7.5 cm in diameter and 5.0 cm in depth were utilized. Throughout this experiment and subsequent ones, the mesh size (2 mm) was sufficiently large to permit unobstructed root emergence from the baskets. The baskets were subsequently filled with soil devoid of fertilizer, and their lower portions were submerged in a hydroponic solution to facilitate normal root development. This method effectively identified genetic loci that contributed to the enhancement of rooting depth and, consequently, increased root dry weight in deeper soil layers under upland field conditions (Uga et al. 2011).

#### 3.2.2 Sand Pots

Plastic pots containing acid‐rinsed, solid‐phase buffered sand or silica sand are subjected to irrigation with hydroponic solutions that encompass variable nutrient concentrations, aimed at investigating the effects on root growth and morphological development (Liu et al. 2023; Zhu and Lynch 2004). This system demonstrates a moderate throughput, as the washing of sand is both efficient and less time‐intensive. Furthermore, modifications to the nutrient composition can be implemented with relative ease.

### 3.3 Potting Mix and Soil Medium

#### 3.3.1 2D Root Growth and Imaging

##### 3.3.1.1 Rhizotrons

**FIGURE 4:**
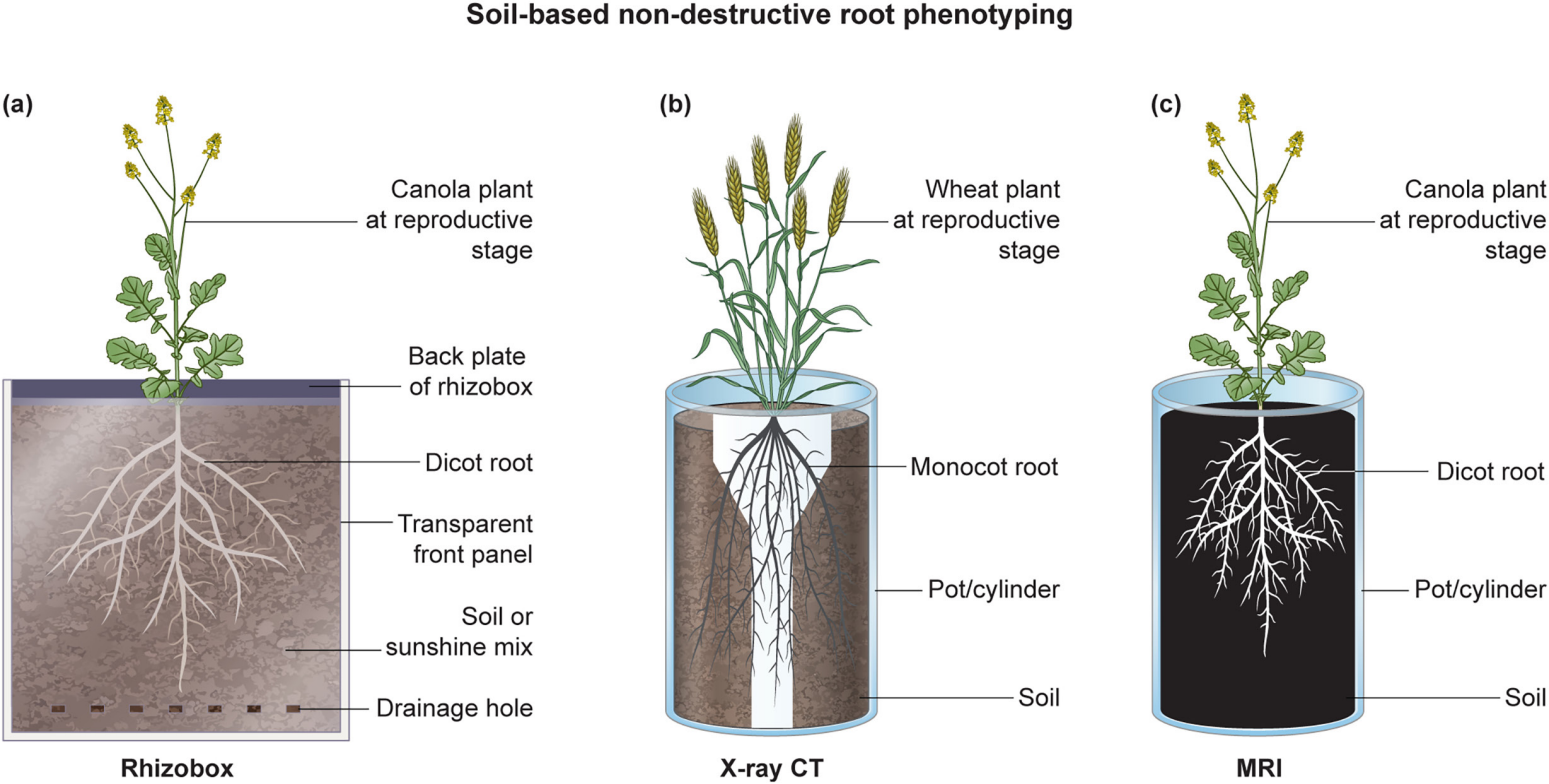
(a) Illustration of a canola plant at the reproductive stage growing in a rhizobox system. (b) Illustration of an x‐ray CT image of the root system architecture of a wheat plant at the heading stage. (c) Illustration of an MRI image of the root system architecture of a canola plant reproductive stage.

Rhizotrons are soil/potting mix‐filled chambers with a transparent window for imaging root systems over time (Figure 4a). They are ideal for assessing vertical 2D root growth and responses to soil layers of varying moisture or compaction (Bontpart et al. 2020; Kuchenbuch and Ingram 2002a; Nagel et al. 2012). Although rhizotron systems enable repeated, non‐destructive visualization of root growth, they still face limitations such as variable root‐zone temperatures, disrupted soil structure, and limited soil or growth medium volume for roots to grow near the transparent rhizotron window (Kuchenbuch and Ingram 2002b; Mooney et al. 2012). Minirhizotrons with built‐in RGB or infrared sensors have advanced high‐throughput root phenotyping by allowing non‐destructive, in situ imaging of root systems in field conditions. They capture detailed structural and physiological traits through multispectral capabilities, combining visible RGB for architecture visualization and infrared for tissue health and water content detection. For example, the SoilCam system, a low‐cost automated minirhizotron, uses a CMOS sensor with customizable IR/UV filters and LEDs at wavelengths like 850 nm (Rahman et al. 2020). It captures 360° panoramic images at up to 2500 DPI inside rigid‐wall tubes. Tested on canola roots, it addressed issues like blur and distortions through post‐processing, enabling time‐lapse monitoring and multispectral analysis to differentiate between living and dead roots (Rahman et al. 2020). Semantic segmentation on RGB mini‐rhizotron images from diverse species uses U‐Net models with EfficientNet encoders, achieving Dice coefficients up to 0.72 and *R* ^2^ of 0.96 for root length, with data augmentation improving generalization (Shen et al. 2020).

#### 3.3.2 3D Soil Phenotyping

##### 3.3.2.1 X‐Ray‐Computed Tomography

X‐ray‐computed tomography (CT) provides high‐resolution three‐dimensional (3D) root architecture in the default growth medium, which is soil (Figure 4b). It is extensively utilized for the analysis of root characteristics, including angles, distribution, and convex hull, among others, offering a 3D spatial and temporal configuration in a dynamic manner within undisturbed soil. The advancements in the imaging resolution of micro/nano CT make it the most precise 3D root phenotyping methodology in natural soil mediums, as it has the capability to capture even fine roots within this opaque environment (Mooney et al. 2012; Rogers et al. 2016; Tracy et al. 2010).

##### 3.3.2.2 Magnetic Resonance Imaging

Magnetic resonance imaging (MRI) exploits the magnetic moment of atomic nuclei, particularly ^1^H protons in living tissues, mainly water (Figure 4c). Strong magnetic and radio frequency fields manipulate this moment to generate 3D datasets. High‐quality images require minimal ferro‐magnetic particles. Contrast parameters like proton density and microenvironments highlight sample differences. This method distinguishes the “root water signal” from the “soil water signal,” providing significant contrast between roots and soil (Metzner et al. 2015; Rogers and Bottomley 1987). MRI offers significant advantages over x‐ray‐computed tomography for assessing dynamic processes like hydration in moist soils. While x‐ray CT provides high spatial resolution for structural imaging, it struggles with low contrast in wet environments and cannot quantify water content. In contrast, despite its lower spatial resolution, MRI enables dynamic and quantitative analyses of water dynamics in root systems. However, MRI tends to be more expensive, less accessible, and requires specialized non‐metallic equipment (Metzner et al. 2015; van Dusschoten et al. 2016).

## 4 Imaging Technologies in Root Phenotyping

Advanced imaging technologies play a pivotal role in non‐destructive root phenotyping, facilitating rapid, repeatable, and scalable assessments of root morphology and physiological status. Two frequently utilized technologies are RGB imaging and hyperspectral imaging, each presenting distinct advantages and limitations depending on the trait of interest and the experimental context.

### 4.1 RGB Imaging

**FIGURE 5:**
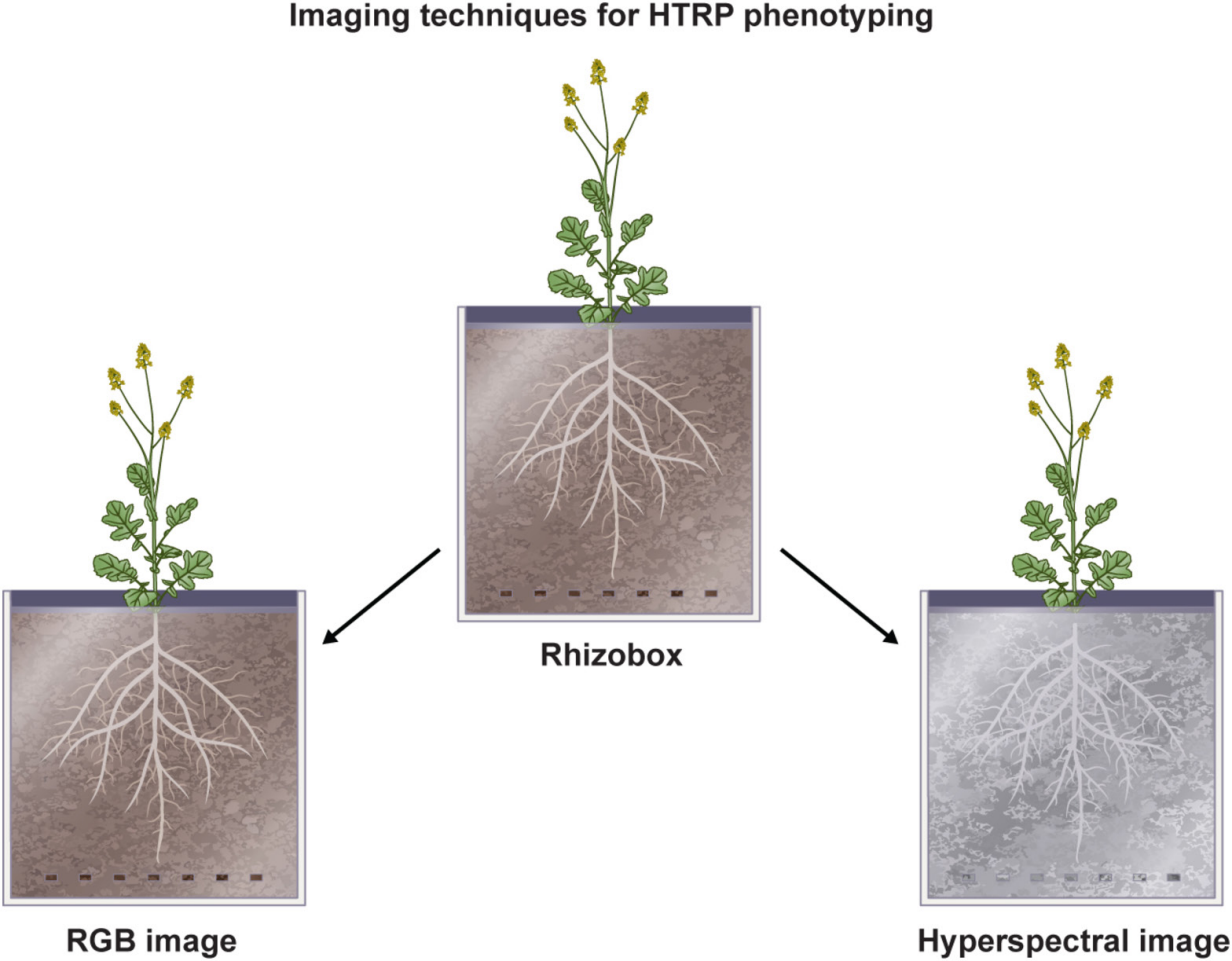
Illustration of RGB and hyperspectral images of a canola plant at the reproductive stage, growing in a Rhizotron system.

Red‐Green‐Blue (RGB) imaging is a widely utilized technique due to its simplicity, cost‐effectiveness, and accessibility (Figure 5). This method captures visible light images and is frequently employed in two‐dimensional and three‐dimensional root phenotyping platforms. It is characterized by low implementation costs, making it suitable for basic laboratory or field settings. The technique offers high spatial resolution, which facilitates clear visualization of root length, branching patterns, angles, and growth rates. Furthermore, it is easily compatible with high‐throughput analysis when integrated with automated root image analysis software. Additionally, it necessitates lower data storage compared to spectral imaging methods. However, it is limited to surface‐level root traits. The method does not provide the capability to detect physiological or biochemical changes within the roots and exhibits reduced performance under conditions where the contrast between roots and background is inadequate, particularly in soil systems.

### 4.2 Hyperspectral Imaging

Hyperspectral imaging (HSI) collects data across numerous narrow spectral bands within the visible, near‐infrared (NIR), and shortwave infrared (SWIR) regions (Figure 5). Unlike RGB images, which are limited to human‐visible light, hyperspectral cameras can identify biochemical signatures in plant tissues. This technology enables the detection of physiological traits such as water status, nutrient levels, and early signs of stress. It can differentiate between healthy and stressed root tissues by analyzing spectral reflectance patterns (Antony et al. 2025; Bodner et al. 2018; Faehn et al. 2024; Marzougui et al. 2019). HSI provides a non‐destructive and multivariate analysis, allowing researchers to investigate the relationships between spectral traits and different genotypes or stress treatments. However, the high costs associated with equipment and data processing infrastructure, along with the need for specialized data analysis tools and expertise, can be limiting factors. Additionally, the data is resource‐intensive, demanding significant storage and computing capabilities. HSI has been employed in hydroponics systems to identify early indicators of early signs of salinity stress (Antony et al. 2025).

## 5 Computational Tools and Data Analysis

### 5.1 Computer Vision‐Based Image Processing Software for Root Analysis

**FIGURE 6:**
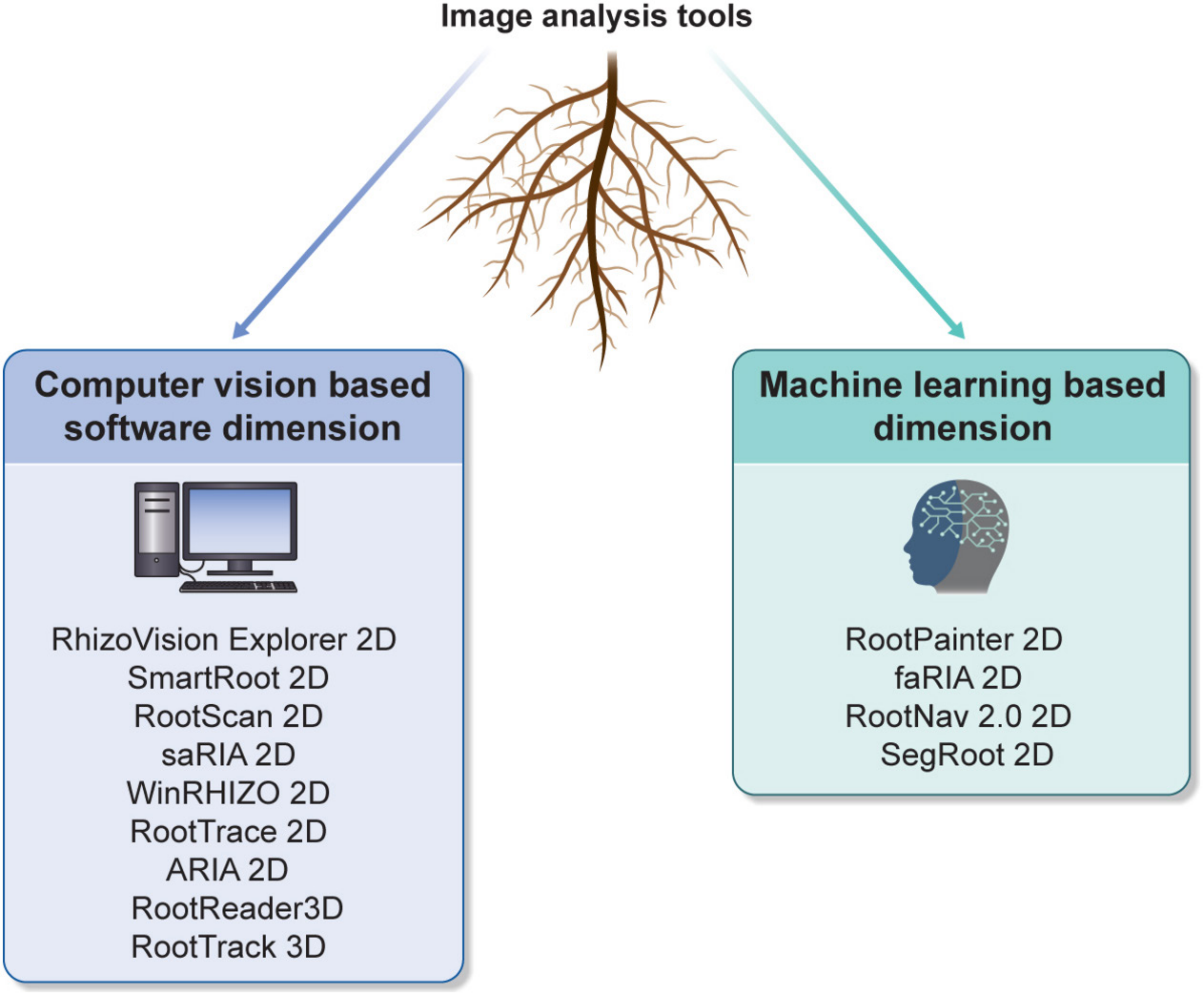
Illustration of commonly used computer vision and machine learning‐based two‐dimensional and three‐dimensional root image analysis software. These are not all, but only a subset of all the resources available for high‐throughput image analysis.

Advanced image processing software tools have revolutionized root phenotyping by enabling the high‐throughput analysis of RSA from 2D or 3D images (Figure 6, Table 1). These tools apply computer vision algorithms for segmenting roots from complex backgrounds, measuring traits such as root length, diameter, angle, depth, and branching patterns, and providing digital models of root growth and development.

**TABLE 1:** Advanced image processing software tools for high‐throughput root system architecture analysis.

Softwares	Journal articles	Root traits measured	Automations	Dimensions
RhizoVision Explorer	Seethepalli et al. (2021)	Length, diameter, area, volume, branching frequency, and root tips (binning by diameter)	Automatic (thresholding, skeletonization, distance mapping using OpenCV)	2D
SmartRoot	Lobet et al. (2011)	Root length, diameter, angle, architecture	Semi‐automatic (user selects/traces roots in ImageJ plugin)	2D
RootScan	Burton et al. (2012)	Cross‐section areas: stele, cortex, aerenchyma, xylem vessels, and cortical cell count	Semi‐automatic (pixel thresholding with GUI for editing regions)	2D
saRIA	Narisetti et al. (2019)	Total length, local width, projection area, volume, spatial distribution, and orientation	Semi‐automatic (adaptive thresholding + user adjustments)	2D
WinRHIZO	Regent Instruments Inc. (Commercial Software)	Root length, surface area, volume, diameter, number of tips, and branching patterns	Automatic (requires scanner setup; software processes root scans)	2D
ARIA	Pace et al. (2014)	Root growth rate, curvature, bending angle, and lateral root density	Automatic (machine learning + image segmentation)	2D
RootTrace	French et al. (2009)	Root length, growth dynamics, curvature, and lateral root number and angles	Semi‐automatic (thresholding and user‐guided tracing)	2D
RootTrack	Mairhofer et al. (2012)	Root system architecture, root elongation rate, angle, length, and dynamics	Automatic (using x‐ray CT images and tracking algorithms)	3D
RootReader3D	Clark et al. (2011)	3D root system architecture: length, angle, depth, width, and volume	Semi‐automatic (user‐guided segmentation from CT or MRI slices)	3D

### 5.2 Machine Learning and AI for Automated Trait Extraction

Machine learning (ML) and artificial intelligence (AI) are increasingly utilized to automate the extraction of traits from complex root image datasets (Figure 6, Table 2). Deep learning models, such as convolutional neural networks (CNNs), can be trained to identify root structures, segment images, and classify architectural traits or stress responses with a high degree of accuracy (Yasrab et al. 2019). These techniques facilitate the rapid processing of extensive imaging datasets and support high‐throughput root phenotyping in both controlled and field environments. Furthermore, AI‐enhanced root image analysis platforms have emerged to address the challenges associated with manual processing and segmentation. Each platform's suitability is reported in Table 3.

**TABLE 2:** Applications of machine learning and artificial intelligence in automated root trait extraction from image datasets.

Tools	Journal article	Root traits	Automatic/semi‐automatic	2D/3D
RootPainter	Smith et al. (2022)	Root length and root nodule counting	Semi‐auto training; automatic after training	2D
faRIA	Narisetti et al. (2021)	Length and area	Fully automatic	2D
RootNav 2.0	Yasrab et al. (2019)	Length, root count, and angles (RSML output)	Fully automatic	2D
SegRoot	Wang et al. (2019)	Length	Automatic	2D

**TABLE 3:** Comparison of root high‐throughput phenotyping methods.

Phenotyping platform	Cost	Throughput	Root stage compatibility	Data dimensionality	Required computational support
Free‐floating hydroponic systems	Low	High	Seedling to mature	2D	Low to medium (image processing)
2D pouches and gel plates	Low	High	Primarily seedling	2D	Low (simple image analysis)
RhizoTubes	Medium	High	Seedling to adult	2D	Medium (image analysis)
3D hydroponic systems	Low to medium	Medium	Seedling to mature	3D	Medium (3D reconstruction)
3D Gellan gum and transparent soil systems	Medium	Low to medium	Seedling	3D	High (3D imaging and analysis)
Basket method	Low	Medium	Mature	2D	Low
Sand pots	Low	Medium	Seedling to mature	3D	Low to medium
Rhizotrons	Medium	Medium	Seedling to mature	2D	Medium (time‐lapse imaging)
Minirhizotrons with RGB or multispec sensors	Medium to high	Low to medium	Seedling to Mature	2D	Medium (image analysis)
x‐ray computed tomography	High	Low to medium (advances enabling higher)	Seedling to mature	3D	High (segmentation software)
Magnetic resonance imaging	High	Low	Seedling to mature	3D	High
RGB imaging	Low	High	Seedling to mature	2D (RGB channels)	Low to medium (computer vision)
Hyperspectral imaging	Medium to high	Medium	Seedling to mature	2D (high spectral bands)	High (spectral analysis)

## 6 Successful Case Studies for Different Abiotic Stresses

Advanced root phenotyping technologies have been instrumental in identifying root traits associated with tolerance to nitrogen (N) and phosphorus (P) deficiencies. For instance, across various plant species, the cell cycle employs a “pause and play” mechanism during recovery from environmental stresses, allowing roots to halt division amid abiotic challenges (such as salt, osmotic, cold, or heat) and restart it for optimal adaptation (Hazelwood et al. 2025).

### 6.1 Nutrient Deficiency (Phosphorus Deficiency Tolerance)

In rice ( *Oryza sativa* L.), Phosphorus uptake1 (Pup1) is a significant quantitative trait locus associated with phosphorus deficiency tolerance, provided by the aus‐type Indian variety, Kasalath. This locus was located on the long arm of chromosome 12 (Heuer et al. 2009; Ni et al. 1998; Wissuwa et al. 2002, 1998). Near‐isogenic lines containing the Kasalath allele at Pup1 demonstrated three times greater phosphorus uptake and grain yield in low‐phosphorus trials compared to the recurrent parent, cultivar Nipponbare, which is sensitive to phosphorus shortage (Wissuwa and Ae 2001). Following detailed mapping of Pup1, comparative sequence analyses of homologous bacterial artificial chromosomes revealed that a genomic segment from Kasalath harbored several genes absent in cultivar Nipponbare, underscoring a roughly 90‐kb deletion in the Nipponbare reference genome that includes the Pup1 locus (Uga et al. 2009). Within this insertion/deletion, OsPupK46‐2, a gene coding for a Ser/Thr kinase from the Receptor‐like Protein Kinase LRK10L‐2 subfamily, was identified as enhancing grain yield and phosphorus uptake in rice lines overexpressing this gene, indicating its role in the Pup1 locus (Gamuyao et al. 2012). Now known as PHOSPHORUS‐STARVATION TOLERANCE1 (OsPSTOL1), OsPupK46‐2 was found to be upregulated in the root tissues of tolerant near‐isogenic lines under phosphorus‐deficient conditions, indicating its potential to increase phosphorus uptake through a physiological mechanism that promotes early root growth and development. Additionally, lines overexpressing OsPupK46‐2 exhibited an approximate 30% increase in grain yield compared to the null lines, suggesting that PSTOL1 could be valuable for molecular breeding to enhance crop performance under low phosphorus conditions. This aligns with the proposed physiological mechanism of OsPSTOL1, where the superior performance of the transgenic lines was associated with improved root dry weight, root length, and root surface area (Gamuyao et al. 2012).

### 6.2 Drought (Root Depth Ratio)

Oyanagi et al. (1993) introduced the mesh‐basket technique for the study of wheat ( *Triticum aestivum* L.). They positioned small hemispherical wire baskets filled with soil surrounding seedlings, categorizing roots based on their extent of penetration into the basket, thereby effectively measuring the angular distribution of emerging roots. This “basket method” enabled the prediction of rooting depth based on root angles. In a subsequent investigation, Nakamoto and Oyanagi (1996) employed a similar design, cultivating wheat seedlings in small hemispherical mesh baskets to accurately evaluate the angles of primary and lateral seminal roots (ϕ and θ). Their findings underscored significant genotypic differences in root angle and spacing, thereby demonstrating the practicality of mesh‐basket assessments in controlled environments. These foundational studies, particularly those conducted by Oyanagi et al. (1993) and Nakamoto and Oyanagi (1996), established the groundwork for subsequent research by Kato et al. (2006), who filled hemispherical baskets with soil surrounding rice plants and calculated the ratio of roots located above versus below a 45° angle to determine root growth angle and deep rooting ratio. Kato et al. (2006) measured genetic variation in root growth angle across 12 cultivars by evaluating the proportion of roots surpassing 50°. Uga et al. (2009) expanded this methodology to 59 rice accessions, again calculating root depth ratio (RDR) within a mesh basket and observing considerable variability, with no clear Japonica/Indica grouping. Uga et al. (2011) employed basket‐derived RDR data from 117 IR64 × Kinandang Patong RILs to map a major quantitative trait locus (DRO1 on chromosome 9), which explained about 66% of the variation in deep rooting, defined as the proportion of roots penetrating the lower half of the basket. In conclusion, the basket assay presents a straightforward, quantitative method for assessing root growth angle and deep rooting in rice, thereby facilitating the phenotyping of seedlings for QTL analysis related to deep‐root traits.

### 6.3 Metal Toxicity (Aluminum‐Tolerant Root Growth)

Ninamango‐Cárdenas et al. (2003), Maron et al. (2008) and Maron et al. (2010) utilized a high‐throughput free‐floating hydroponic phenotyping platform to investigate the genetic factors that contribute to aluminum (Al) tolerance in maize ( *Zea mays* L.). By cultivating seedlings in nutrient solutions containing controlled and toxic levels of Al, they effectively quantified root growth traits, particularly relative root elongation, as a measure of Al tolerance. This platform enabled meticulous, large‐scale screening of various genotypes and mapping populations. Through QTL analysis, Ninamango‐Cárdenas et al. (2003) identified several loci associated with Al tolerance, including a significant QTL on chromosome 6. The transcription profiling study conducted by Maron et al. (2008) identified a significantly differentially expressed gene, ZmMATE1, from the MATE family, which co‐localized with the Al tolerance QTL on chromosome 6. Expression analysis demonstrated that ZmMATE1 is significantly upregulated in tolerant lines under Al stress. Notably, Maron et al. (2010) discovered that increased tolerance was correlated with copy number variation; the most tolerant maize lines possessed multiple tandem copies of ZmMATE1, resulting in heightened transcript levels and citrate efflux.

### 6.4 Cold Stress (Cold‐Tolerant Root Growth)

Qin and Chandnani et al. (in preparation; personal communication Dr. Leon Kochian) conducted high‐throughput root phenotyping for cold‐tolerant root growth in the soybean association panel at the seedling stage. Uniformly germinated soybean seedlings from a very early maturity group soybean diversity panel were transplanted in a hydroponic solution maintained at 10°C, and a parallel experiment was conducted at the control temperature. Results showed significant genetic variation in cold‐tolerant root growth. Consequently, in the field validation study, a significant positive correlation was observed between seedling cold‐tolerant root growth and root growth phenotyped in field conditions by shovelomics.

### 6.5 Salt Stress (Salt‐Tolerant Root Growth)

The tolerance to salt stress in narrowleaf trefoil ( *Lotus tenuis* Waldst. & Kit. ex Willd.) and tall fescue ( *Festuca arundinacea* ) is intricately associated with specific root architectural adaptations that enhance resource acquisition in saline conditions. In tall fescue, genotypes that exhibit salt tolerance demonstrate an increased total root length, deeper primary roots, and elongated lateral roots, which collectively contribute to improved water and nutrient uptake in challenging soil environments (Fan et al. 2023). Simultaneously, narrowleaf trefoil exhibits a distinctive herringbone lateral root configuration, which facilitates efficient exploration of the soil and supports continued nitrogen fixation, even under conditions of salt stress (Paz et al. 2012). These root traits are fundamental in enabling both species to sustain growth and productivity in saline environments.

## 7 Bridging the Gap Between Controlled‐Environment High‐Throughput Root Phenotyping and Field Conditions

High‐throughput root phenotyping conducted in controlled environments such as growth chambers or hydroponic systems facilitates precise measurement of root traits, including architecture, growth rates, and responses to specific stressors. However, it often fails to capture the complex soil heterogeneity, microbial interactions, and environmental variability characteristic of field settings (Watt et al. 2013). This discrepancy can limit the translational applicability of laboratory findings to real‐world agricultural contexts, where factors such as soil compaction, nutrient gradients, and weather fluctuations significantly influence root performance. Recent advancements emphasize the integration of field‐based methods with laboratory‐derived insights to improve predictive accuracy for crop breeding and stress resilience.

Shovelomics serves as a vital field‐to‐laboratory integration technique, enabling high‐throughput excavation and visual assessment of root crowns from mature plants cultivated in natural soil conditions (Trachsel et al. 2011). Initially developed for maize, it quantifies traits such as root angle, number, and density within the top 20–30 cm of soil, providing rapid phenotypic data that correlate with architectural traits measured in laboratory settings. Developments in digital imaging platforms, such as DIRT (Digital Imaging of Root Traits), automate the analysis of excavated roots by utilizing supercomputing resources to extract over 75 traits, thereby bridging manual field sampling with computational phenotyping (Das et al. 2015). Recent applications in wheat and legumes demonstrate its utility in multi‐site trials, where field‐excavated roots are imaged and correlated with controlled‐environment data to validate genetic markers associated with drought tolerance (Burridge et al. 2016; York et al. 2018).

The Lee Hickey research team at the University of Queensland has pioneered the application of unmanned aerial vehicle (UAV) imaging technology to deduce the correlations between canopy and root systems (Alahmad et al. 2025). Using multispectral drones, researchers capture vegetation indices (VIs) that act as indicators for belowground traits. Their research on barley shows that canopy biochemical composition, measured through VIs at key growth stages, can accurately predict root biomass and architecture. This helps close the gap between laboratory and field studies by non‐invasively linking aboveground phenotypes to root features. This study combined UAV data with machine learning to indirectly examine genetic variation in root systems, reaching correlations of up to 0.70 between canopy traits and root depth under field stress. Additionally, recent research highlights the use of non‐destructive x‐ray‐computed tomography in paddy fields, complementing UAV methods by enabling the detection of quantitative trait loci and conducting genome‐wide association studies (GWAS) for rice root traits without excavation (Teramoto and Uga 2025).

Digital twins, through 4D x‐ray CT modeling, create virtual replicas for trait extraction and growth simulations, enhancing predictions of resource uptake and yield under diverse scenarios, though root‐specific applications remain emerging (Herrero‐Huerta et al. 2021).

Layering rhizosphere microbiome data combines microbial community profiles with root phenotyping, demonstrating translational progress. High‐resolution sampling uncovers how different root zones attract specific microbes for nutrient cycling, and metagenomic networks reveal pathogen suppression through microbiome amendments (Xu et al. 2021). Recent developments include genome‐resolved metagenomics connecting iron metabolism to drought resilience in plant‐microbe interactions (Xu et al. 2021). In bioenergy crops, integrating microbiome data with root traits improves phenotyping for sustainable production, as supported by York et al. (2022).

## 8 Challenges and Limitations

**FIGURE 7:**
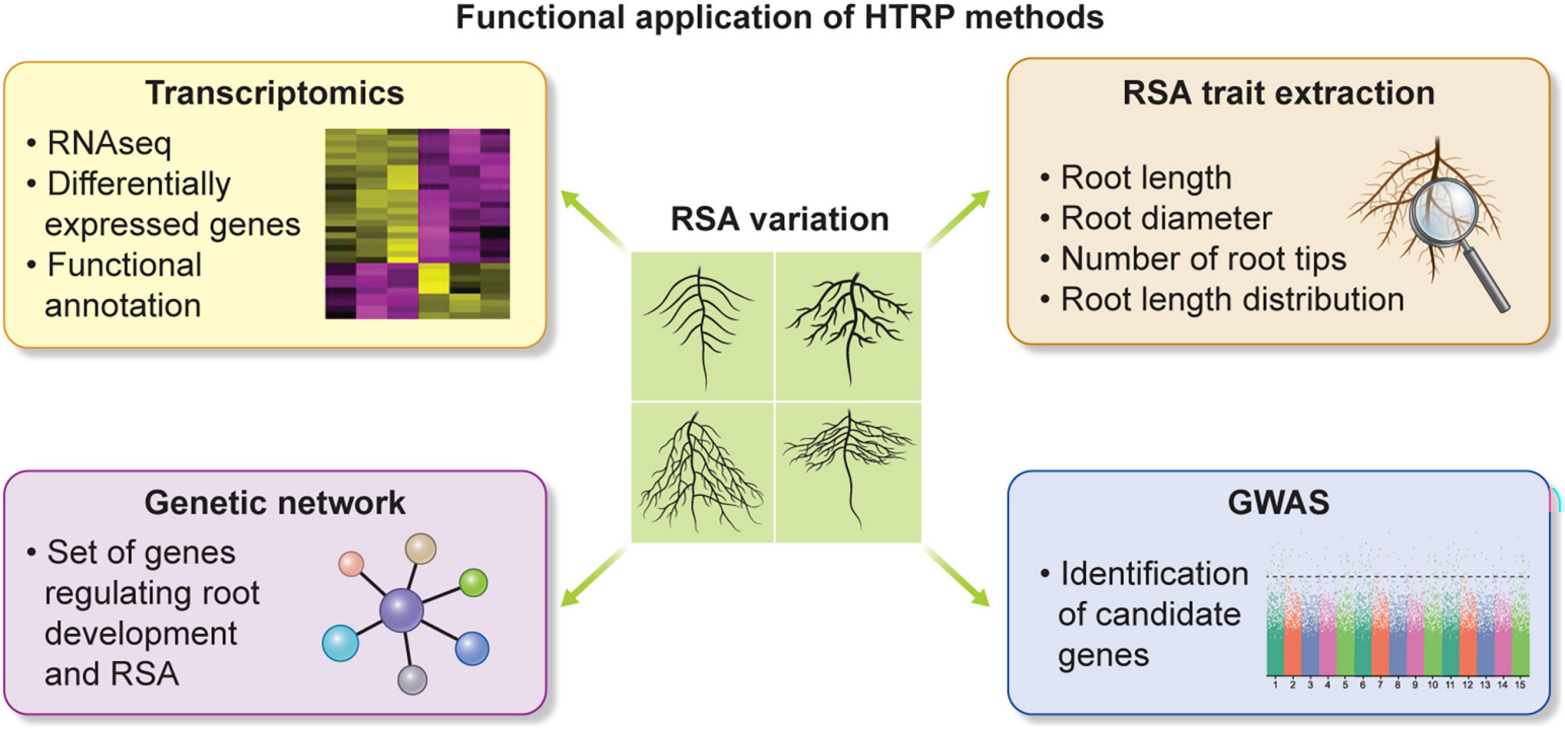
Illustration of a workflow exhibiting functional application of the high‐throughput root phenotyping systems in the identification and functional characterization of genetic variants shaping root system architecture in model and economic crops.

In various studies, high‐throughput root phenotyping along with quantitative trait mapping, association mapping, and functional genetics has identified root development and root trait regulation genes (Figure 7). High‐throughput root phenotyping technologies, which are designed to rapidly analyze plant root systems on a large scale, encounter significant limitations that impede their complete effectiveness and applicability. Imaging challenges, including the incapacity of non‐invasive techniques such as MRI, CT, or x‐ray to capture fine root structures or deep roots due to resolution constraints and soil opacity, are exacerbated by soil heterogeneity, which obscures roots amid varying textures, moisture levels, and organic debris. Numerous systems depend on 2D imaging because of the highest throughput, which oversimplifies complex 3D root architectures, whereas controlled environments such as hydroponics or gel‐based setups do not replicate field conditions, thereby limiting insights into root‐soil interactions that are critical for real‐world agriculture. Research indicates that hydroponic systems used in high‐throughput root phenotyping may miss genotype‐environment (G × E) interactions due to their uniform conditions. This can result in weak correlations, often below 0.50, between root traits in the lab and those in the field, although the strength of these correlations varies depending on the trait and crop (Kuijken et al. 2015; Langstroff et al. 2022; Liu et al. 2017). Multi‐environment trials evaluate RSA plasticity across stress gradients. For instance, maize research demonstrates adaptive responses to drought, nitrogen deficiency, and salinity, mainly through deeper roots (Keerthi et al. 2025). This evidence suggests that crops such as maize adopt adaptive RSA responses under stress conditions, often by developing deeper roots. However, the complexity of G × E interactions emphasizes the importance of balanced breeding strategies across different environmental stakeholders. Similarly, hydropatterning is a genetically complex trait that guides lateral root branching towards moisture, enhancing water uptake in heterogeneous soils. In maize, growth‐maintained water potential gradients influence this patterning process via auxin signaling (Robbins and Dinneny 2018). Research indicates that post‐translational modifications of ARF7 affect branching, promoting development on water‐contact sides; meanwhile, SUMOylation inactivates ARF7 on air sides (Orosa‐Puente et al. 2018). Tropical maize germplasm lines show stronger responses associated with genetic loci related to auxin, ethylene, and arabinogalactan proteins, highlighting potential breeding targets for drought tolerance (Scharwies et al. 2025).

The extensive datasets generated necessitate advanced computational tools and expertise for image processing and trait quantification; however, automated segmentation algorithms frequently misidentify roots in noisy images, and the absence of standardized protocols complicates cross‐study comparisons. High costs and specialized infrastructure hinder access to advanced platforms, particularly for smaller research groups, while time‐intensive setup and processing can slow down large‐scale studies. Most systems concentrate on structural traits such as root length or branching but encounter challenges in measuring functional traits like nutrient uptake or microbial interactions, often neglecting genotype‐environment interactions, which diminishes the robustness of findings. Invasive methods pose a risk of damaging delicate roots, non‐invasive methods lack detail, and capturing temporal dynamics remains challenging, as most platforms provide snapshots rather than continuous monitoring. Furthermore, integrating root data with aboveground traits such as shoot growth or yield proves difficult, thereby limiting the holistic insights required for breeding programs.

## Author Contributions

R.S. conceptualized the research project and secured funding. R.C. and R.S. jointly designed the manuscript framework and developed the illustrative figures. R.C. drafted the manuscript with input from R.S. Both authors reviewed and approved the final version of the manuscript.

## Supporting information


**Table S1:** Abbreviations glossary.

## Data Availability

Data sharing is not applicable to this article as no new data were created or analyzed in this study.
