# Supplementary material for: The Invisible Frontline: High‐Tech Root Imaging for Crop Stress Adaptation
Source: Physiol Plant. 2025 Oct 15;177(5):e70572. doi: 10.1111/ppl.70572 (PMC12528975; doi:10.1111/ppl.70572)
Supplement: Supplementary file 1 — Table S1: Abbreviations glossary. [file PPL-177-e70572-s001.docx]

**Supplementary Table 1. Abbreviations glossary**

| **Abbreviation** | **Description** |
| --- | --- |
| AI | Artificial intelligence |
| CNN | Convolutional neural networks |
| CT | Computed tomography |
| DIRT | Digital Imaging of Root Traits |
| G×E | Genotype by environment interaction |
| GWAS | Genome-wide association studies |
| HSI | Hyperspectral imaging |
| HTRP | High-throughput root phenotyping |
| MET | Multi-environment trials |
| ML | Machine learning |
| MRI | Magnetic resonance imaging |
| NIR | Near-infrared |
| QTL | Quantitative trait loci |
| RBG | Red-Green-Blue |
| RSA | Root system architecture |
| SWIR | Shortwave infrared |
| UAV | Unmanned aerial vehicle |
| VI | Vegetation indices |
